# Frequent Versus Single Active Bouts Differentially Affect Movement Behavior and Energy Balance in Adults With Overweight/Obesity

**DOI:** 10.1002/oby.70310

**Published:** 2026-10-06

**Authors:** Heloisa C. Santo Andre, Elisa Le Roux, Nathan P. De Jong, Patricia R. Smith, Andrew H. Lange, Carlos Mendez, Alexandre Zahariev, Melissa L. Mamele, Ginger Johnson, Zhaoxing Pan, Chantal Simon, Daniel H. Bessesen, Ana J. Pinto, Audrey Bergouignan

**Affiliations:** ^1^ Division of Endocrinology, Metabolism and Diabetes, Anschutz Health and Wellness Center, Department of Medicine, School of Medicine University of Colorado Anschutz Medical Campus Aurora Colorado USA; ^2^ Faculty of Applied Sciences State University of Campinas Limeira São Paulo Brazil; ^3^ Institut Pluridisciplinaire Hubert Curien, UMR7178 Centre National de la Recherche Scientifique, Université de Strasbourg Strasbourg France; ^4^ Section of Endocrinology, Department of Pediatrics, School of Medicine University of Colorado Anschutz Medical Campus Aurora Colorado USA; ^5^ CarMeN Laboratory INSERM 1060, INRA 1397, University Claude Bernard Lyon 1, Human Nutrition Research Center Rhône‐Alpes Oullins France

**Keywords:** accelerometry, appetite, doubly labeled water, energy expenditure, humans

## Abstract

**Objective:**

This study aimed to investigate the effects of breaking up sedentary behavior (SB) on daily movement behavior and energy balance in adults with overweight/obesity.

**Methods:**

Thirty participants (16F/14M; 34.2 ± 7.3 years; 29.5 ± 3.2 kg/m^2^) were randomized to either BREAK (multiple 5‐min brisk walking bouts) or a duration‐matched intervention, ONE (45‐min brisk walking), performed 5 days/week for 6 weeks. At pre‐ and post‐intervention, daily SB and physical activity (PA; accelerometry), body composition (doubly labeled water [DLW]), total daily energy expenditure (TDEE; DLW), appetite, and fasting leptin were measured. Linear mixed‐effects models tested time effects and group‐by‐time interactions.

**Results:**

Only BREAK reduced prolonged SB (interaction: *p* = 0.043). Both groups shifted SB‐PA composition toward greater moderate‐to‐vigorous PA, with proportional reductions in SB and light PA (time: all *p* ≤ 0.011). These changes in PA level were associated with increased TDEE (time: *p* = 0.040). ONE showed small increases in body and fat mass compared with BREAK (interaction: both *p* ≤ 0.061). No differences were noted in metabolizable energy intake, appetite, or leptin levels.

**Conclusions:**

Spreading short PA bouts throughout the day increases moderate‐to‐vigorous PA and TDEE to the same extent as a traditional continuous PA bout. Future studies should investigate whether the modest body composition changes observed across interventions are driven by distinct behavioral/physiological compensations influenced by the daily pattern of PA/SB.

**Trial Registration:**

ClinicalTrials.gov: NCT02998892

## Study Importance

- What is already known?
  ○ Acutely, breaking up prolonged sedentary behavior (SB) with short bouts of physical activity (PA) reduces food cravings compared to a single continuous PA bout.
  ○ Single continuous PA bouts have been associated with compensatory reductions in non‐exercise activities (daily living activities) in some studies, which may attenuate increases in total daily energy expenditure (TDEE) and limit effects on body mass and adiposity.
- What does this study add?
  ○ Performing brisk walking as either frequent short bouts or a single continuous bout over 6 weeks increased MVPA and TDEE to a similar extent; however, only frequent short bouts reduced prolonged sedentary time (> 60 min).
  ○ Despite no differences in metabolizable energy intake, appetite, or plasma leptin, the single continuous bout was associated with borderline, small increases in body and fat mass compared with short bouts, suggesting possible differential compensatory adaptations.
- How might these results change the direction of research or the focus of clinical practice?
  - Promoting frequent short bouts of PA throughout the day can improve daily movement and help meet current PA/SB guidelines to a similar extent as traditional PA strategies while also reducing prolonged SB.
  - Future studies are needed to investigate the influence of PA accumulation patterns on long‐term energy balance and body composition and whether short, frequent bouts of PA differentially influence the compensatory adaptations often observed with exercise (MVPA) training.

## 1 Introduction

Sedentary behavior (SB), characterized by low energy expenditure (≤ 1.5 METS) in sitting, reclining, or lying down positions, is an independent risk factor for chronic conditions, including insulin resistance, type 2 diabetes, and obesity [1]. In Westernized countries, direct assessments indicate that adults spend ~8–12 h/day sedentary [2, 3]. Higher SB is associated with greater body mass index (BMI), waist circumference, and total and trunk fat mass [4, 5].

Breaking up SB with short bouts of physical activity (PA) has been associated with lower body mass and waist circumference, independent of overall SB or moderate‐to‐vigorous PA (MVPA) levels [6]. A meta‐analysis including data from 33 studies conducted in adults with normal weight or overweight/obesity reported modest reductions in body mass, waist circumference, and body fat percentage following SB reduction interventions [7].

Body mass loss reflects cumulative negative energy balance over time, i.e., total daily energy expenditure (TDEE) exceeding energy needs. Acutely, 1‐ to 5‐min bouts of low‐intensity walking increase energy expenditure compared with uninterrupted sitting and standing breaks to SB [8, 9]. We showed that spreading moderate‐intensity PA over 6 h reduced pre‐meal food cravings compared with a single 30‐min bout in healthy adults [10]. Similarly, replacing sitting time (−21%) with standing reduced appetite and energy intake in office workers over 4 weeks [11]. These findings suggest that breaking up SB may increase TDEE while decreasing energy intake, supporting a more favorable energy balance. However, no study has examined the impact of active breaks on energy balance over extended periods. Furthermore, it remains unclear whether the pattern of PA accumulation itself influences energy balance beyond the effect of increasing total MVPA.

This 6‐week study aimed to compare the effects of breaking up SB with frequent short PA bouts versus a single PA bout matched for total active time (45 min/day, 5 days/week) on daily SB/PA, body mass and composition, and energy balance components. We hypothesized that spreading regular active breaks would more effectively reduce prolonged SB and limit spontaneous behavioral adaptation (i.e., decreases in light PA [LPA]) than performing a single continuous bout, thereby promoting a more favorable energy balance.

## 2 Methods

### 2.1 Study Design

This was a single‐center, parallel‐group randomized controlled trial (RCT; Figure 1). Potentially eligible participants were invited for a screening visit, which included a medical H&P examination, a fasting blood draw, the International Physical Activity Questionnaire (IPAQ) to assess habitual PA, and a 5‐day assessment of habitual step count using a pedometer. Habitual SB/PA, TDEE, resting metabolic rate (RMR), appetite, and leptin concentration were measured at baseline (Pre) and at the end of the interventions (Post).

**FIGURE 1:**
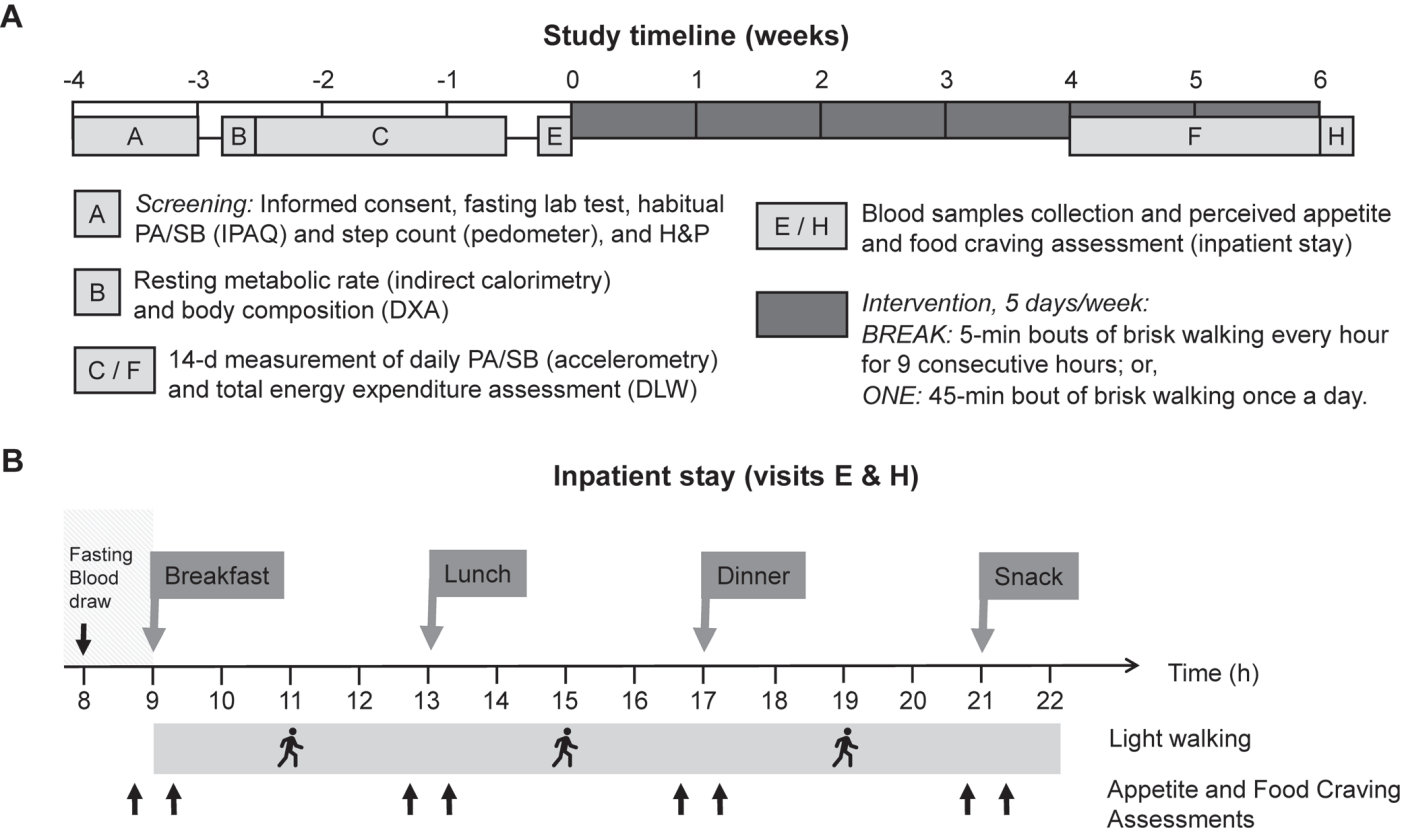
Study design overview. DLW, doubly labeled water; DXA, dual‐energy X‐ray absorptiometry; H&P, health and physical examination; IPAQ, International Physical Activity Questionnaire; PA, physical activity; RMR, resting metabolic rate; SB, sedentary behavior.

### 2.2 Ethics Statement

This study was approved by the Colorado Multiple Institutional Review Board (COMIRB; protocol 16‐1769) and conducted in accordance with the Declaration of Helsinki [12]. Written informed consent was obtained from each participant.

### 2.3 Participants

Eligible participants were 18–45 years old with BMI 25–38 kg/m^2^, weight stable for > 6 months, sedentary (> 6 h/day self‐reported sitting), and physically inactive (< 150 min/week of self‐reported MVPA and/or < 6500 steps/day). Exclusion criteria included insulin resistance, diabetes, cardiovascular, renal, or hepatic disease, uncontrolled hypertension, cancer, HIV, substance abuse (drugs, tobacco, or > 40 g/day alcohol), psychiatric disorders, medications affecting lipid/energy metabolism, PA contraindications, and pregnancy/lactation/post‐menopause. Participants were recruited from May 2017 to October 2021 in the Denver metro area (Colorado).

### 2.4 Intervention

Participants were randomized (1:1), stratified by sex, to either [1] ONE, moderate‐intensity brisk walking for 45 min, 5 days/week; or [2] BREAK, ten 5‐min bouts of brisk walking every hour, 5 days/week. In BREAK, ten bouts were prescribed rather than nine to account for the approximately 10% lower completion of prescribed short‐bout MVPA previously observed under free‐living conditions [13], thereby aiming to ensure ~45 min/day of completed activity at the prescribed intensity. This adjustment was pragmatic and was not intended to address behavioral compensation differently across interventions. All participants completed a treadmill familiarization session, beginning at 2.0 mph (3.2 km/h) with 0.3 mph (0.5 km/h) increments every 2 min, to develop an understanding of the target moderate‐intensity effort (i.e., a Borg rating of 6 [“somewhat hard”] out of 10), and this perceived exertion level was used to guide the intensity of the PA bouts. Because the intervention was performed under free‐living, unsupervised conditions, absolute exercise intensity during the prescribed bouts was guided by individualized perceived exertion rather than standardized treadmill speed, workload, or %HRmax. Fitbit step cadence data were used to monitor adherence. The statistician generated the allocation sequence and placed it into numbered opaque envelopes. Outcome assessors and researchers were not blinded.

The intervention lasted 5.7 (mean) ± 1.9 weeks. Adherence was monitored using a wrist‐worn Fitbit Charge 2 (San Francisco, CA), worn continuously throughout the intervention (Figure S1). Participants were considered adherent if > 80% of prescribed PA was achieved (i.e., ≥ 36 min/day of continuous PA in ONE and ≥ 7 bouts/day in BREAK; ≥ 4 days/week for both). To support compliance, participants tracked progress via the Fitbit app and received regular motivational messages or individualized follow‐ups. To maximize adherence, participants were allowed to perform the activity at their preferred time of day, whether fasted or fed. They were instructed to maintain their habitual diet and avoid intentional weight loss; however, dietary intake during the intervention was not monitored.

### 2.5 SB and Physical Activity

Participants wore an ActivPAL4 (right thigh; PAL Technologies, Glasgow, Scotland) and an ActiGraph GT3X+ (right hip; Ametris, Fort Walton Beach, FL) for 14 consecutive days at baseline and during the final two intervention weeks. The ActiGraph was removed during sleep and water‐based activities. A daily log captured sleep and non‐wear time, which was used to correct wear time (ActiGraph) and waking time (activPAL). Criteria for valid data for both devices were **≥** 10 h/day on ≥ 4 days, including ≥ 1 weekend day [14, 15]. Mean wear time for ActiGraph was 13.6 ± 1.2 h/day. Mean waking time for activPAL was 15.7 ± 1.1 h/day.

ActiGraph GT3X+ quantifies PA (inactivity, LPA, and MVPA). Data were cleaned (e.g., non‐wear time identification, removal of invalid days), analyzed, and exported using ActiLife6 software, v.6.11.9 (Ametris, Pensacola, FL). Freedson cut points were used to estimate inactivity (< 100 cpm), LPA (≥ 100 to < 1952 cpm), and MVPA (≥ 1952 cpm) [16]. Inactivity primarily refers to SB but also includes standing or minimal movement.

ActivPAL4 assesses postural allocation (sitting, standing, and stepping). Data were cleaned (e.g., sleep time adjustment, removal of invalid days), analyzed, and exported using PALanalysis software v.7.2.32 (PAL Technologies), using PAL Technologies' proprietary posture‐allocation algorithm. These metrics of SB were computed: total SB (time spent sitting, in seated transport, and secondary lying down), total breaks in SB (number of times a sitting/lying down event was followed by a standing or stepping event), and time in prolonged SB bouts ≥ 60 min (sum of time spent in SB bouts ≥ 60 and < 120, ≥ 120 and < 240, and ≥ 240 min). Total time spent standing and stepping and daily step count were computed. Because sleeping time was removed from the analysis, SB corresponds to waking SB.

### 2.6 Total Daily Energy Expenditure and Its Components

TDEE was measured using the doubly labeled water (DLW) method over 14 days, as previously described [17]. Subjects ingested a premixed 2 g/kg of estimated total body water (TBW) dose of DLW composed of 0.2 and 0.15 g/kg of 10% enriched H_2_^18^O and 99% enriched ^2^H_2_O (CIL, Andover, MA), respectively [18, 19]. Urine samples were collected in duplicates every other day for the determination of ^2^H and ^18^O enrichment by isotope ratio mass spectrometry (Delta V Advantage IRMS, Thermofisher Scientific, UK). Details on equilibration and endpoint urine cleaning, mass spectrometry procedures, quality check controls, and calculations are published elsewhere [20]. TDEE was calculated using Speakman's equation [21], with individual 24‐h respiratory quotients measured during the 24‐h whole‐room calorimeter stays at baseline and post‐intervention.

Activity energy expenditure (AEE) was calculated as AEE_(MJ/day)_ = TDEE_(MJ/day)_—diet‐induced thermogenesis (DIT)_(MJ/day)_—RMR_(MJ/day)_. RMR was measured via indirect calorimetry with a metabolic cart (ParvoMedics TrueOne 2400, Salt Lake City, UT) for 20 min after an overnight fast at rest [22]. DIT was assumed to be 10% of TDEE. PA level (PAL) was calculated as participants' TDEE divided by their RMR.

### 2.7 Body Composition

Body mass was measured using a calibrated scale. At baseline, body composition was measured by dual‐energy X‐ray absorptiometry (DXA; Hologic Delphi‐W, Bedford, MA) [23]. Changes in body composition were estimated by isotopic dilution. Fat‐free mass from DLW was calculated by estimating a hydration coefficient of 73.2%, and fat mass was calculated as the difference between body mass and fat‐free mass [18, 19]. The agreement between the DXA and hydrometry data at baseline was 0.26 kg (−2.05 to 2.58) for fat‐free mass (*r* ^2^ = 0.99, *p* < 0.001).

### 2.8 Metabolizable Energy Intake

Metabolizable energy intake was calculated using the principle of energy balance [24, 25] as: Energy intake _(MJ/day)_ = TDEE_(MJ/day)_ + ΔES_(MJ/day)_. Changes in energy store (ΔES) were calculated using the individual body composition change predicting model [26]:

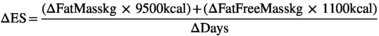




### 2.9 Perceived Appetite and Food Cravings

During Pre and Post 24‐h inpatient visits, participants received a standardized diet (55% carbohydrate, 30% fat, and 15% protein of energy intake) calculated to match their energy needs. Meals were provided at 0900, 1300, 1700, and 2100 h (30%, 30%, 30%, and 10% of daily energy intake). Individual energy needs were estimated as RMR × activity factor, with RMR derived from the average of measured values (indirect calorimetry) and DXA‐based estimates ([23.9 × fat‐free mass in kg] + 372) [27, 28]. The activity factor was based on average PAL values from previous studies conducted under similar inpatient conditions. Energy balance during the inpatient stay was assessed as the difference between total energy expenditure measured over 24 h and the energy intake provided by the University of Colorado Clinical and Translational Research Center metabolic kitchen.

Participants rated hunger and prospective food consumption before meals and fullness at 1 h post‐meal, using a visual analog scale (VAS). The Food Cravings Questionnaire—State (15 items; 1–5 scale) was completed before each meal [29]. Total score reflects craving intensity; subscales [30] assessed desire to eat, anticipated reward, relief from negative states, loss of control, and physiological craving (hunger). Meal scores were averaged to obtain daily values.

### 2.10 Fasting Leptin Concentrations

On the day of the 24‐h inpatient visits, an intravenous catheter was inserted into an antecubital vein for a blood draw. Plasma leptin concentrations were measured using an ultra‐sensitive ELISA kit (Ultrasensitive Leptin ELISA Kit, ALPCO, NH, USA; 22‐LEPHUU‐E01; intra‐assay CV: 2.63%–4.35%; inter‐assay CV: 6.1%–7.5%) in the Beckman Coulter AU480 Chemistry Analyzer (Brea, CA, USA).

### 2.11 Statistical Analysis

Participant characteristics were summarized using descriptive statistics.

The primary inferential focus was on the group‐by‐time interaction, expressed as the estimated mean difference in change between groups (EMD; ΔBREAK–ΔONE) with 95% confidence intervals (95% CI). When the interaction was not statistically significant, the main effect of time was examined and reported as the estimated time difference (ETD; Post–Pre) with 95% CI.

All dependent variables, except for step count, were analyzed using linear mixed models (LMM) with group (BREAK vs. ONE) and time (Pre vs. Post) as fixed factors and participant as a random factor, using a compound symmetry (CS) covariance structure.

Time‐dependent variables for daily PA and SB were analyzed using a compositional approach, as previously described [31]. Movement behaviors were expressed as three‐part compositions (ActiGraph: inactivity, LPA, and MVPA; activPAL: SB, standing, and stepping) and transformed using isometric log‐ratio (ilr) coordinates. Two ilr coordinates were derived per system (e.g., ilr1 representing inactivity relative to LPA and MVPA, ilr2 representing the balance between LPA and MVPA), and the ilr system was rotated to allow interpretation of each behavior individually. Only ilr1 is reported in the main text, as it captures the primary contrast of interest, whereas ilr2 estimates and stacked LMM including both ilr coordinates and a balance factor were explored as sensitivity analyses to assess internal redistribution of behaviors.

Daily step count was analyzed using a generalized LMM with group, time, and their interaction as fixed factors, participant as a random factor, a negative binomial distribution with log link, and wear time included as an offset to account for exposure and overdispersion.

All analyses were adjusted for sex, given its known influence on energy balance components and movement behavior. Analyses were also adjusted for other covariates depending on the outcome: waking time for sit‐to‐stand transitions; body mass for TDEE and its components; squared height for body mass, fat mass, and fat‐free mass to account for differences in body size; body mass and energy balance (calculated as the difference between 24‐h TDEE measured from gas exchange in the whole‐room calorimeter and measured energy intake [27]) for appetite and food cravings; and body mass for fasting leptin. Analyses adjusted for sex only are presented in the online Supporting Information.

Analyses followed the intention‐to‐treat (ITT) principle, with missing data handled using LMM via restricted maximum likelihood estimation under the assumption that data were missing completely at random or missing at random. The sample sizes of valid data for each outcome are described in Table S1, along with the reasons for missing data.

LMM residual diagnostics were assessed graphically (Q–Q plots and residuals vs. fitted values) to evaluate normality and homoscedasticity of conditional residuals, and influential observations were examined using studentized residuals. For generalized LMM with a negative binomial distribution, model adequacy was assessed using appropriate residual and overdispersion diagnostics.

Variables were analyzed on their original scale unless a transformation was required to satisfy model assumptions. Appetite, food cravings, and fasting leptin were Blom‐transformed due to non‐normal distributions of model residuals.

Sensitivity analyses were conducted by excluding extreme observations and two participants who deviated from the protocol (i.e., one exceeded the prescribed PA dose, and another one unexpectedly traveled during Post data collection). Results from sensitivity analyses that differ from ITT are discussed.

Data analysis was performed using SAS 9.4 (SAS Institute Inc., Cary, NC). The study statistician was not blinded to group allocation. Data are presented as mean ± standard deviation (SD), median (25th, 75th percentile), or least square means (LSMeans) with 95% CI, as appropriate. For compositional data, model estimates (LSMeans) obtained on ilr coordinates were back‐transformed to the original scale for interpretation. Briefly, ilr coordinates were rescaled, exponentiated, and normalized to obtain compositional proportions expressed in min/day or percentages. Back‐transformed ETD and EMD represent multiplicative changes over time and multiplicative between‐group differences in change, respectively. No adjustment for multiple comparisons was applied. Significance was set at *p* ≤ 0.05 for main effects. Because interaction tests are generally less powerful than tests of main effects and given the proof‐of‐concept nature and limited sample size of this study, we prespecified a threshold of *p* < 0.10 for interaction terms to reduce the risk of type II error. However, findings based on this threshold should be considered exploratory.

## 3 Results

### 3.1 Participants' Characteristics

The CONSORT diagram is shown in Figure S2. Thirty participants were randomized to BREAK (*n* = 8F/7M) or ONE (*n* = 8F/7M) groups. Participants had a mean age of 34.2 ± 7.3 years, BMI of 29.5 ± 3.2 kg/m^2^, and adiposity of 37.8% ± 7.9%. Baseline HOMA‐IR was 1.5 ± 0.6, indicating participants did not have insulin resistance (Table 1).

**TABLE 1:** Participant characteristics.

	BREAK (*n* = 15)	ONE (*n* = 15)
Sex, *n* [%]		
Female	8 [53.3%]	8 [53.3%]
Male	7 [46.7%]	7 [46.7%]
Age (years)	33.7 ± 7.6	34.7 ± 7.3
Race, *n* [%]		
White	10 [66.7%]	10 [66.7%]
Black	4 [26.7%]	2 [13.3%]
Other	1 [6.7%]	3 [20.0%]
Ethnicity, *n* [%]		
Hispanic	2 [13.3%]	6 [40.0%]
Non‐Hispanic	13 [86.7%]	9 [60.0%]
Body mass (kg)	87.0 ± 15.5	84.2 ± 10.1
Height (cm)	170.0 ± 8.6	169.8 ± 9.4
BMI (kg/m^2^)	29.9 ± 4.2	29.1 ± 1.9
Fat‐free mass (kg)	53.0 ± 10.0	52.0 ± 10.3
Fat mass (kg)	33.1 ± 11.0	31.1 ± 6.3
Fat mass (%)	38.0 ± 8.6	37.7 ± 7.5
HOMA‐IR	1.6 ± 0.5	1.5 ± 0.7
RMR (kcal)	1584.3 ± 246.9	1592.6 ± 232.0
5‐day average daily steps	4639 ± 1026	4867 ± 1185
Self‐reported sitting (h/day)	10.6 ± 2.8	10.5 ± 2.8

*Note:* Data presented as mean ± SD or *n* [%]. 5‐day average daily steps measured by pedometer during the screening period.

Abbreviation: RMR, resting metabolic rate.

### 3.2 Both Interventions Increased MVPA and Reduced Sedentariness, but Only BREAK Reduced Prolonged Sitting

Table 2 summarizes PA/SB outcomes adjusted for sex. Participants' adherence to the prescribed PA protocols was high, with no significant difference between BREAK and ONE (94.3% ± 15.0% vs. 96.6% ± 9.4%; *p* = 0.606).

**TABLE 2:** Habitual sedentary behavior and physical activity level.

Outcomes	BREAK (*n* = 15)		ONE (*n* = 15)		ETD (95% CI)	EMD (95% CI)	Group effect, *p*	Time effect, *p*	Interaction effect, *p*
	Pre	Post	Pre	Post					
Intervention									
Adherence to prescribed PA (%)	—	94.3 ± 15.0	—	96.6 ± 9.4	—	—	0.606	—	—
Intervention duration (weeks)	—	5.7 ± 2.1	—	5.7 ± 1.8	—	—	0.963	—	—
ActivPAL^a^									
Total SB (min/day)	631.2 (565.9, 696.6)	602.8 (542.0, 663.7)	644.7 (581.6, 707.8)	596.5 (536.5, 656.5)	0.80 (0.67, 0.94)	1.03 (0.74, 1.45)	0.453	0.011	0.842
Compositional mean (min/day [%])	637.5 (69.6%)	609.4 (65.9%)	654.7 (68.3%)	607.4 (63.1%)					
Standing time (min/day)	205.2 (164.4, 246.1)	211.6 (174.6, 248.7)	212.7 (175.2, 250.2)	235.7 (192.0, 279.3)	0.96 (0.87, 1.06)	0.91 (0.75, 1.11)	0.678	0.357	0.341
Compositional mean (min/day [%])	200.1 (21.9%)	207.8 (22.5%)	206.4 (21.5%)	228.3 (23.7%)					
Stepping time (min/day)	79.3 (64.2, 94.4)	109.8 (87.4, 132.2)	101.5 (80.9, 122.1)	131.0 (103.8, 158.2)	1.31 (1.16, 1.48)	1.07 (0.84, 1.36)	0.096	< 0.001	0.581
Compositional mean (min/day [%])	78.1 (8.5%)	107.2 (11.6%)	97.8 (10.2%)	127.1 (13.2%)					
Prolonged SB ≥ 60 min (% total SB)^b^	34.3 (28.8, 39.8)	26.5 (21.0, 32.0)	29.5 (24.0, 35.0)	30.1 (24.4, 35.7)	−3.6 (−7.7, 0.4)	−8.4 (−16.5, −0.3)	0.855	0.079	0.043
Sit‐to‐stand transitions (number/day)^b^	44.4 (39.5, 49.3)	45.4 (40.5, 50.3)	50.7 (45.8, 55.5)	48.5 (43.5, 53.5)	−0.6 (−3.5, 2.4)	3.1 (−2.8, 9.0)	0.144	0.701	0.288
Step count (number/day)^b^	6477 (5427, 7729)	9559 (8003, 11,419)	7490 (6269, 8949)	10,611 (8839, 12,739)	1.45 (1.25, 1.68)	1.04 (0.78, 1.40)	0.221	< 0.001	0.778
ActiGraph^a^									
Inactivity time (min/d)	577.0 (546.1, 608.0)	567.5 (527.7, 607.3)	542.5 (484.2, 600.7)	546.6 (480.6, 612.6)	0.68 (0.52, 0.89)	0.87 (0.51, 1.49)	0.669	0.007	0.600
Compositional mean (min/day [%])	584.7 (72.3%)	580.3 (70.4%)	547.4 (67.6%)	555.3 (67.5%)					
Light‐intensity PA (min/day)	211.0 (183.8, 238.2)	206.3 (173.4, 239.2)	249.0 (199.2, 298.7)	242.6 (185.3, 299.8)	0.65 (0.53, 0.79)	0.95 (0.64, 1.42)	0.117	< 0.001	0.803
Compositional mean (min/day [%])	208.3 (25.8%)	205.4 (24.9%)	247.8 (30.6%)	236.1 (28.7%)					
Moderate‐to‐vigorous PA (min/day)	20.9 (13.2, 28.6)	50.8 (25.7, 75.9)	18.3 (12.0, 24.6)	33.5 (23.1, 43.9)	2.24 (1.50, 3.33)	1.24 (0.56, 2.74)	0.461	< 0.001	0.589
Compositional mean (min/day [%])	15.7 (1.9%)	38.8 (4.7%)	14.6 (1.8%)	31.3 (3.8%)					

Abbreviations: EMD, estimated mean difference; ETD, estimated time differences; PA, physical activity; SB, sedentary behavior.

^a^ Data are presented as mean (95% CI) for raw movement composition values and as estimated compositional mean (percentage of total composition), obtained by back‐transforming isometric log‐ratio coordinates from LMM. Estimated time difference (ETD) refers to the exponentiated main effect of time contrast, representing the multiplicative change in the relative balance between behaviors over time (Post–Pre). ETD reflects shifts in the relative composition of movement behaviors rather than absolute min/d; therefore, changes in larger components may appear proportionally larger than their absolute change. ETD > 1 indicates an increase in a behavior relative to the remaining composition, whereas ETD < 1 indicates a decrease in its relative dominance. Estimated mean difference (EMD) refers to differences between changes in BREAK and in ONE (ΔBREAK—ΔONE), which represents the multiplicative difference in compositional change between interventions over time. EMD > 1 indicates greater relative change in BREAK vs. ONE, whereas EMD < 1 indicates smaller relative change or greater decrease in BREAK vs. ONE.

^b^ Data presented as LSMeans (95% CI), ETD (95% CI), and EMD (95% CI), calculated by LMM. ETD refers to changes from Pre to Post in both groups (Post—Pre), whereas EMD refers to differences between changes in BREAK and in ONE (ΔBREAK—ΔONE). All LMM adjusted for sex. Sit‐to‐stand transitions and step count models also adjusted for waking time. For all variables, the sample sizes are *n* = 15 in both groups. *Note:* Step count was measured with ActivPAL over a 14‐day period.

A decrease in the percentage of total SB spent in prolonged bouts (> 60 min) was observed in BREAK (within‐group change: −7.8%, 95% CI: −13.5 to −2.2), whereas no difference was observed in ONE (within‐group change: 0.6%, 95% CI: −5.2 to 6.4; interaction: *p* = 0.043). However, none of the interventions modified sit‐to‐stand transitions (time: *p* = 0.701; interaction: *p* = 0.288). No other time‐by‐group interactions were noted for any PA/SB outcomes, including the compositional changes (interaction: all *p* ≥ 0.34).

Daily step count increased in both groups by 45% (ETD fold change: 1.45, 95% CI: 1.25 to 1.67; time: *p* < 0.001). Both interventions increased time spent in MVPA (ETD: 2.24, 95% CI: 1.50 to 3.33; time: *p* < 0.001), with absolute estimates rising from 15.7 to 38.8 min/day in BREAK and from 14.6 to 31.3 min/day in ONE. This greater dominance of MVPA was accompanied by proportional reductions in inactive time (ETD: 0.68, 95% CI: 0.52 to 0.89; time: *p* = 0.007) and time spent in LPA (ETD: 0.65, 95% CI: 0.53 to 0.79; time: *p* < 0.001) within the composition, although absolute changes were modest. Stepping time also increased (ETD: 1.31, 95% CI: 1.16 to 1.48; time: *p* < 0.001), with absolute estimates rising from 78.1 to 107.2 min/day in BREAK and from 97.8 to 127.1 min/day in ONE. This greater dominance of stepping was accompanied by a proportional reduction in total SB time (ETD: 0.80, 95% CI: 0.67 to 0.94; time: *p* = 0.011), but not standing time (ETD: 0.96, 95% CI: 0.87 to 1.06; time: *p* = 0.357). Results from ilr2 and stacked models confirmed that the patterns of change observed in ilr1 were consistent with the internal redistribution of behaviors, and conclusions were unchanged. Raw ilr1 and ilr2 coordinates are reported in Table S2. Sensitivity analyses yielded the same results as ITT analyses.

### 3.3 BREAK and ONE Significantly Increased TDEE to a Similar Extent

Energetics data were adjusted for body mass and sex. No group × time interactions were observed for energy outcomes (all *p* > 0.74). There was a significant overall increase in TDEE (ETD: +0.67 MJ/day, 95% CI: 0.03 to 1.31; time: *p* = 0.040; interaction: *p* = 0.652; Figure 2A) that was driven by slight, nonsignificant increases in AEE in both groups (ETD: +0.51 MJ/day, 95% CI: −0.04 to 1.05; time: *p* = 0.069; interaction: *p* = 0.537; Figure 2B). RMR did not significantly change in either group (ETD: +0.10 MJ/day, 95% CI: −0.06 to 0.26; time: *p* = 0.200; interaction: *p* = 0.736; Figure 2C). Although not significant, PAL tended to increase in both groups (time: *p* = 0.065; interaction: *p* = 0.504; Figure 2D). Sensitivity analyses yielded similar results. Data adjusted for sex only are available in Table S3.

**FIGURE 2:**
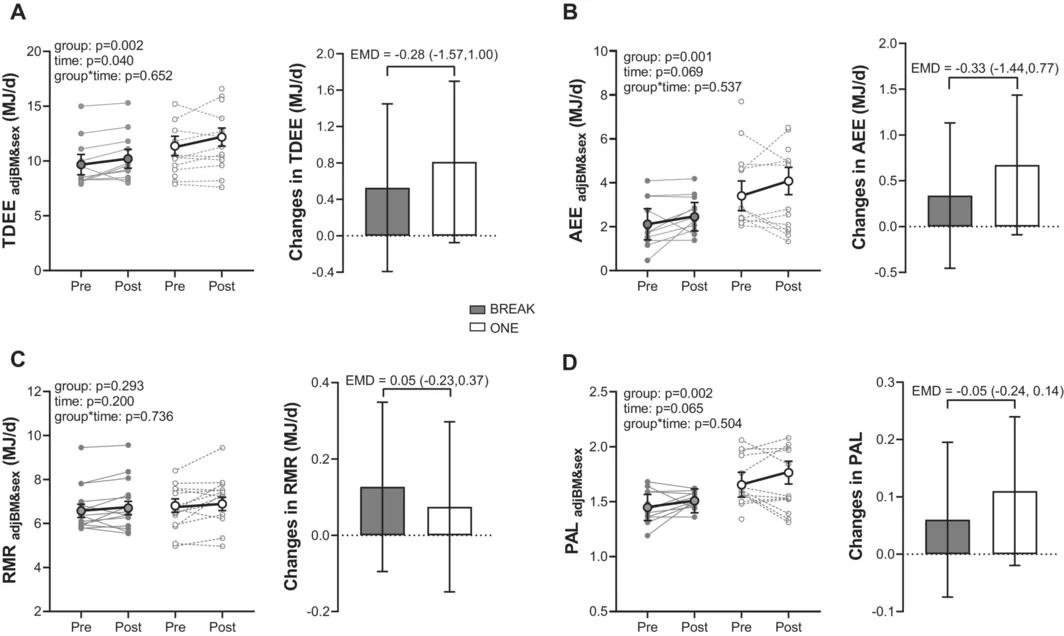
Energy expenditure and its components changes during the protocol. (A) Changes in total daily energy expenditure (TDEE). (B) Changes in activity energy expenditure (AEE). (C) Changes in resting metabolic rate (RMR). (D) Changes in physical activity level (PAL). Data are presented as individual values and LSMeans (95% CI) for graphs on the left and as within‐group changes (95% CI) along with EMD (95% CI) for graphs on the right. Estimated mean difference (EMD) refers to differences between changes in BREAK and in ONE (ΔBREAK–ΔONE). LSMeans and EMD are derived from linear mixed models (LMM) that included group, time, and group‐by‐time as fixed effects and study participants as random effects, with CS as the covariance structure; LMM were adjusted for sex and body mass. For TDEE, AEE, and PAL, the sample size is *n* = 13 for BREAK and *n* = 14 for ONE. For RMR, the sample sizes are *n* = 15 in both groups.

### 3.4 Minor Increase in Body Mass and Fat Mass Following ONE but Not BREAK

Body mass and composition data were adjusted for height squared. A group × time interaction was observed for body mass (*p* = 0.061; Figure 3A), reflecting an increase in ONE (+ 1.5 kg, 95% CI: 0.35 to 2.58), but no change in BREAK (−0.1 kg, 95% CI: −1.2 to 1.1). A similar interaction was observed for fat mass (ONE: +2.4 kg, 95% CI: 0.5 to 4.3; BREAK: −0.2 kg, 95% CI: −1.2 to 1.6; interaction: *p* = 0.055; Figure 3B); fat‐free mass was not modified (time: *p* = 0.653; interaction: *p* = 0.727; Figure 3C).

**FIGURE 3:**
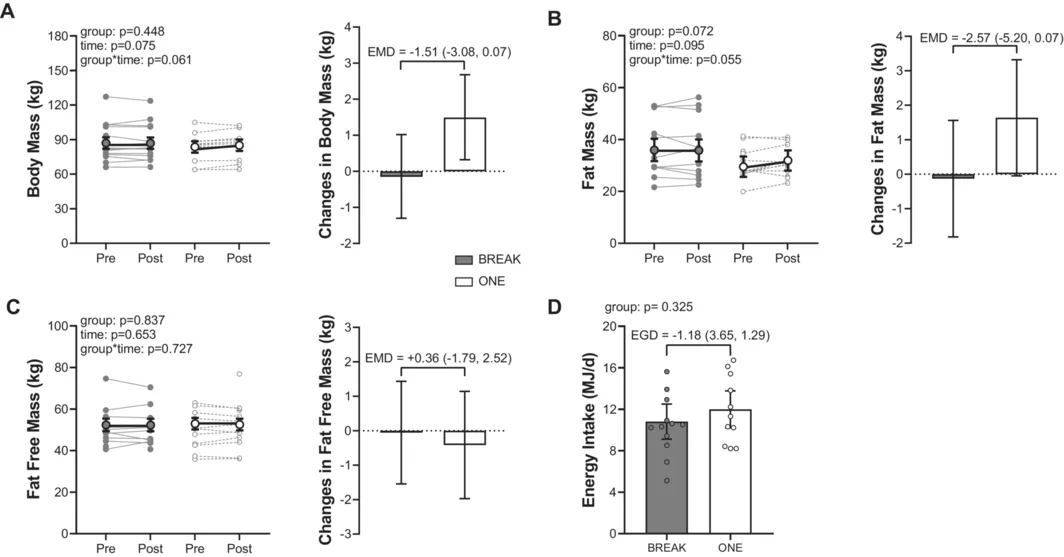
Body mass and composition changes during the protocol and metabolizable energy intake during the interventions. (A) Changes in body mass. (B) Changes in fat mass. (C) Changes in fat‐free mass. (D) Energy intake during the interventions. Data are presented as individual values and LSMeans (95% CI) for graphs on the left and as within‐group changes (95% CI) along with EMD (95% CI) for graphs on the right. Estimated mean difference (EMD) refers to differences between changes in BREAK and in ONE (ΔBREAK—ΔONE). Estimated group difference (EGD) refers to differences between BREAK and ONE (ONE‐BREAK). LSMeans, EMD, and EGD are derived from linear mixed models (LMM) that included group, time, and group‐by‐time as fixed effects and study participants as random effects with CS as the covariance structure; LMM were adjusted for squared height and sex for body mass and body composition and for sex only for estimated energy intake. For body mass, fat‐free mass, and fat mass, the sample size is *n* = 12 in both groups. For energy intake, the sample size is *n* = 10 for BREAK and *n* = 9 for ONE.

Sensitivity analyses excluding the participant who exceeded the prescribed PA dose in BREAK attenuated these interactions (body mass: *p* = 0.116; fat mass: *p* = 0.106) and indicated a significant overall increase over time in body mass (+0.9 kg, 95% CI: −0.2 to 1.6; time *p* = 0.018) and fat mass (+1.4 kg, 95% CI: −0.2 to 2.6; time *p* = 0.026), still driven by the ONE group.

### 3.5 No Effect on Appetite, Food Cravings, Leptin Concentrations, or Metabolizable Energy Intake

Estimated daily metabolizable energy intake, adjusted for sex, during the intervention period did not differ between groups (EGD: −1.18 MJ/day, 95% CI: −3.65 to 1.29; *p* = 0.325; Figure 3D).

Adjusted for body mass, appetite‐ and food cravings‐related scores remained unchanged (all *p* > 0.17; Table 3), as well as fasting leptin concentrations (time: *p* = 0.112; interaction: *p* = 0.928; Table 3). Appetite, food cravings, and leptin data adjusted for sex only are available in Table S3.

**TABLE 3:** Appetite, food cravings, and fasting leptin.

Outcomes	BREAK (*n* = 13)		ONE (*n* = 13)		ETD (95% CI)	Group effect, *p*	Time effect, *p*	Interaction effect, *p*
	Pre	Post	Pre	Post				
Appetite								
Hunger	64.67	67.67	67.83	61.33	−0.33	0.859	0.834	0.282
	(53.00, 81.67)	(59.83, 77.67)	(61.42, 74.92)	(57.67, 70.33)	(−9.00, 7.00)			
Fullness	76.33	70.67	73.00	78.00	0.83	0.778	0.983	0.530
	(63.00, 81.33)	(58.00, 80.67)	(61.67, 81.00)	(64.67, 85.50)	(−6.58, 7.67)			
Prospective consumption	61.67	70.33	65.50	58.00	−1.67	0.149	0.621	0.405
	(54.33, 81.33)	(64.50, 81.33)	(53.25, 72.42)	(53.33, 62.67)	(−9.33, 5.33)			
Food cravings	43.00	47.33	47.00	41.83	0.33 (−6.17, 4.50)	0.555	0.654	0.811
	(40.67, 46.33)	(40.17, 50.50)	(41.92, 51.92)	(38.17, 50.58)				
Desire to eat	8.33	10.00	9.67	8.17	0.00	0.440	0.629	0.716
	(7.33, 9.00)	(7.33, 11.17)	(8.75, 11.08)	(7.42, 10.92)	(−2.00, 1.33)			
Anticipation of positive reinforcement	10.00	10.67	10.50	9.67	−0.33	0.525	0.383	0.405
	(9.00, 11.33)	(8.50, 11.17)	(9.08, 11.17)	(7.83, 10.83)	(−1.67, 1.00)			
Anticipation of relief from negative states	8.67	9.67	9.17	9.17	0.00	0.149	0.621	0.405
	(7.67, 9.33)	(8.50, 10.67)	(8.83, 12.08)	(8.00, 11.58)	(−0.83, 0.83)			
Lack of control overeating	7.33	6.00	6.17	6.17	0.33	0.989	0.971	0.174
	(5.67, 8.00)	(5.17, 7.50)	(6.00, 7.67)	(6.00, 6.92)	(−1.00, 1.67)			
Craving as a physiological state	10.00	11.00	10.67	10.33	0.00	0.565	0.939	0.971
	(9.33, 12.00)	(9.67, 11.33)	(8.83, 12.42)	(9.17, 11.33)	(−1.50, 0.67)			
Fasting leptin (ng/mL)^a^	1.52	1.52	1.61	2.07	−0.09	0.950	0.112	0.928
	(0.93, 2.72)	(0.83, 2.61)	(0.82, 3.01)	(0.62, 2.38)	(−0.33, 0.18)			

*Note:* Data are presented as median (25th, 75th percentiles) and median ETD (25th, 75th percentiles). Estimated time difference (ETD) refers to changes from Pre to Post in both groups (Post–Pre). All appetite and food cravings data are provided in A.U. (arbitrary unit). Appetite and food cravings LMM adjusted for sex, body mass, and energy balance during the inpatient stay. Fasting leptin LMM adjusted for sex and body mass. For appetite and food cravings variables, the sample sizes are *n* = 13 in both groups.

^a^ For fasting leptin concentrations, the sample sizes are *n* = 14 for BREAK and *n* = 15 for ONE.

## 4 Discussion

This is the first RCT to prospectively examine, over several weeks, whether breaking up SB with brief bouts of PA alters daily movement patterns and energy balance differently than a traditional single daily PA bout, matched for intensity and total duration, in sedentary adults with overweight or obesity. Main findings are (i) both interventions increased daily MVPA, step count, and TDEE to a similar extent; (ii) only the BREAK intervention reduced time spent in prolonged SB (≥ 60 min); and (iii) borderline group‐by‐time interactions were observed for body mass and fat mass. These outcomes remained relatively stable in BREAK, whereas small increases were observed in ONE. Although these findings are consistent with the possibility that the pattern of PA accumulation may influence short‐term energy balance regulation, the interactions were attenuated in sensitivity analyses, warranting cautious interpretation.

Many studies have examined the acute and short‐term effects of short active breaks, but very few have tested such interventions over several weeks in free‐living conditions [7, 32]. Here, BREAK reduced the proportion of daily SB accumulated in prolonged bouts from 34.3% to 26.5%, whereas no change was observed in ONE. This distinction is important because prolonged SB has been associated with less favorable cardiometabolic profiles independent of total SB and MVPA [6, 33]. However, although epidemiological studies support the relevance of SB patterns, the dose–response relationship for clinically meaningful benefits remains unknown. Therefore, the reduction in prolonged SB should be viewed as a favorable behavior change, but its clinical significance remains to be established.

Prescribing brisk walking for 45 min/day, 5 days/week, as a single PA bout or as repeated short bouts, increased daily MVPA in sedentary adults with overweight/obesity. Participants in both groups reached MVPA levels (BREAK: 38.8 min/day; ONE: 31.3 min/day) consistent with guidelines (> 30 min/day) [34] and increased step count toward levels associated with lower chronic disease and mortality risk (8000–10,000 steps/day) [35]. This supports the broader public health message: “every movement counts” [34]. Notably, both interventions also reduced SB by 30 min/day, a magnitude relevant to population health [36].

Our findings are also consistent with earlier trials suggesting that accumulating short bouts of PA across the day can be a viable alternative to a single continuous bout for promoting adherence and achieving broadly comparable outcomes. In studies by Jakicic et al., shorter accumulated bouts produced outcomes comparable to longer continuous bouts in sedentary adults with overweight, particularly from an adherence and behavioral perspective [37, 38]. Unlike those earlier trials, however, the present study was not designed primarily as a weight loss intervention, but rather to examine free‐living SB patterns and components of energy balance, which may explain the different emphasis of our findings.

An important observation is that the prescribed MVPA did not simply replace SB. In both groups, increases in MVPA occurred at the expense of sedentary time and LPA, supporting the interdependence of daily movement components. A spontaneous reduction in non‐exercise PA (e.g., daily walking, LPA) and increased sedentary time has been previously reported following exercise (MVPA) training, particularly in sedentary individuals with overweight/obesity [17, 39]. Yet, contrary to our hypothesis [40], spreading PA as short bouts throughout the day did not better preserve LPA than a single continuous session matched for volume and intensity. Thus, while BREAK altered the pattern of sedentary accumulation, it did not produce a clearly different redistribution of overall daily movement composition than ONE. Nonetheless, population data show that replacing LPA with MVPA confers health benefits [41], including reductions in body mass or adiposity [42], suggesting both interventions may improve health.

Like the behavioral data, the energetics data do not support the idea that BREAK reduced overall compensatory responses (decreases in RMR and/or lower‐than‐expected increases in AEE due to spontaneous behavioral adaptations) regularly reported in response to structured MVPA interventions [39, 43]. As described by King et al., these compensatory responses may be driven by physiological fatigue, motivational factors (e.g., perceiving inactivity as a reward), and neuro‐regulatory processes protecting against sustained negative energy balance [44]. Despite a modest reduction in LPA, both interventions increased TDEE to a similar extent, with no evidence of differential effects on AEE or RMR.

Estimated metabolizable energy intake did not differ significantly between groups. Although this approach is likely more robust than self‐reported dietary methods [45], it may still not capture subtle compensatory changes in food intake over time, particularly in the context of small body composition changes and substantial day‐to‐day variation in intake. Likewise, no changes in appetite ratings, food cravings, or fasting leptin were detected. These findings differ from our previous acute study, in which frequent short bouts of moderate‐intensity PA reduced pre‐meal food cravings, whereas a matched continuous bout did not, both compared to a sedentary condition [10]. Several factors may explain this discrepancy, including differences in study design (acute 6‐h vs. 6‐week) and population (individuals with normal weight vs. overweight/obesity) and the fact that appetite was not assessed on days when the intervention itself was performed. It is also possible that appetite responses to altered movement patterns are subtle and influenced by non‐homeostatic drivers of intake (e.g., food reward, palatability, environmental cues) that promote intake beyond homeostatic needs [46, 47], and that they were not captured here. More broadly, individuals with overweight or obesity often exhibit altered appetite regulation and may require stronger or longer perturbations to elicit measurable changes in appetite and food intake.

The small increase in body mass and fat mass observed in ONE, but not in BREAK, is intriguing, as it was not clearly explained by differences in estimated metabolizable energy intake or TDEE. This may reflect a small cumulative energy imbalance that was not detected with the measures used, particularly given the relatively short duration, missing data, or day‐to‐day variability that becomes relevant over longer time periods. Nevertheless, these findings should be interpreted cautiously, as the group‐by‐time interactions were attenuated and no longer met the prespecified threshold in sensitivity analyses excluding an over compliant BREAK participant. Taken together, these data highlight the need for future studies investigating the influence of PA accumulation patterns on long‐term energy balance and body composition and whether short frequent bouts of PA differentially influence the compensatory adaptations often observed with exercise training.

Importantly, this study should be considered a proof‐of‐concept conducted under relatively controlled free‐living conditions. Frequent activity breaks were supported by regular contact and motivational prompts, and the prescribed PA dose (45 min/day, 5 days/week) exceeds minimum recommendations. Therefore, the feasibility and effectiveness of less structured, scalable interventions remain to be established. Larger or more sustained changes in movement behavior may be required to elicit clinically meaningful health benefits.

This study has several strengths, including high adherence, direct PA/SB assessment using complementary devices (ActiGraph, ActivPAL), gold‐standard measurement of free‐living TDEE using DLW, and balanced sex distribution. Limitations include the relatively short duration, which may have limited detection of robust changes in body composition and energy balance components. Additionally, behavioral and energetic compensation may also emerge over longer intervention durations. The use of isotopic dilution rather than repeated DXA is also a limitation, although baseline agreement was strong, in addition to the lack of post‐prandial assessment of appetite‐regulating hormones (e.g., ghrelin, GLP‐1, PYY), which could provide insights given their links to energy compensation and fat mass loss [43, 48]. Although estimated metabolizable energy intake did not provide continuous day‐to‐day dietary tracking, it was derived from objective measures of free‐living energy expenditure and changes in body energy stores. Lastly, sample sizes were relatively small for some outcomes due to technical issues and missing data, including TDEE, body composition, and estimated metabolizable energy intake, which may have limited power to detect small between‐group differences and increase uncertainty around borderline findings. Accordingly, these findings should be interpreted cautiously and warrant confirmation in larger, longer‐term trials.

## 5 Conclusion

Both a single daily bout of brisk walking and frequent short bouts accumulated across the day increased MVPA and TDEE in sedentary adults with overweight or obesity. Only BREAK reduced prolonged sedentary time. Body mass and fat mass remained stable in BREAK and tended to increase modestly in ONE; and future fully powered trials should confirm these findings. Overall, the prescription of short brisk walking bouts to break up sedentary time was associated with more favorable sedentary‐accumulation patterns without compromising TDEE. Longer‐term studies are warranted to determine whether these behavioral changes translate into clinically meaningful health benefits.

## Author Contributions

D.H.B. and A.B. conceived and designed research; A.B. secured funding; N.P.D.J., A.H.L., C.M., D.H.B., and A.B. collected data; H.C.S.A., A.Z., G.J., A.J.P., and A.B. analyzed samples; H.C.S.A., E.L.R., Z.P., C.S., A.J.P., and A.B. analyzed data; H.C.S.A., E.L.R., N.P.D.J., P.R.S., C.M., A.H.L., A.Z., M.L.M., Z.P., C.S., D.H.B., A.J.P., and A.B. interpreted results; H.C.S.A., A.J.P., and A.B. prepared figures; H.C.S.A., A.J.P., and A.B. drafted manuscript. All authors have read, revised, and approved the final version of the manuscript.

## Funding

This work was supported by the National Institute of Diabetes and Digestive and Kidney Diseases of the National Institutes of Health, Grant Numbers R00DK100465, F31DK125061 and P30DK48520 (to A.B.), NIH/NCATS Colorado CTSA, Grant Number UL1 TR001082 (to A.B.), and TL1TR002533 (to N.P.D.J.). This study was also supported by a grant from the São Paulo State Research Support Foundation, Grant Number 2023/17782‐3 (to H.C.S.A.) and by the American Diabetes Association, Grant 1‐25‐PDF‐103 (to A.J.P).

## Conflicts of Interest

D.H.B. receives research funding from Novo Nordisk, Eli Lilly, and Pfizer. The other authors declared no conflicts of interest.

## Supporting information


**Figure S1:** Agreement between research‐grade accelerometers, activPAL (top) and actiGraph (bottom left), and Fitbit monitors (bottom right) for determining adherence to the ONE (Panel A) and BREAK (Panel B) intervention protocols. Panel A depicts an example of a participant who performed the ONE intervention from 8:30 to 9:15. Panel B depicts an example of a participant who performed the BREAK intervention from 11:00 to 21:00.
**Figure S2:** Consolidated Standards of Reporting Trials (CONSORT) flow diagram. Exclusion and missing data for specific outcomes are described in Table S2. BREAK, 9 hourly 5‐min brisk walking bouts, 5 days/week; ONE, single 45‐min brisk walking bout, 5 days/week.
**Table S1:** Participants with complete data and reasons for missing data for all study outcomes.
**Table S2:** Model estimates for isometric log‐ratio (ilr) coordinates of the movement compositions.
**Table S3:** Sex‐adjusted only model estimates for energy expenditure, appetite, food cravings, and fasting leptin.

## Data Availability

The trial protocol, statistical analysis plan, and deidentified datasets generated and analyzed during the current study are available from the corresponding author on reasonable request, via secure file transfer. Data can be shared with researchers for purposes of reproducing the results or conducting meta‐analyses and will be available indefinitely from the time of publication.
